# Novel role of HIV-1 Nef in regulating the ubiquitination of cellular proteins

**DOI:** 10.3389/fcimb.2023.1106591

**Published:** 2023-03-08

**Authors:** Maria Ghaly, Jessica Proulx, Kathleen Borgmann, In-Woo Park

**Affiliations:** Department of Microbiology, Immunology, and Genetics, University of North Texas Health Science Center, Fort Worth, TX, United States

**Keywords:** HIV-1 Nef, post-translational modification, ubiquitinated proteins, ubiquitin, proteasomal degradation system

## Abstract

Our recent data established that HIV-1 Nef is pivotal in determining the fate of cellular proteins by modulating ubiquitination. However, it is unknown which proteins are ubiquitinated in the presence of Nef, a question critical for understanding the proliferation/restriction strategies of HIV-1 in infected cells. To identify cellular proteins ubiquitinated by Nef, we conducted a proteomic analysis of cellular proteins in the presence and absence of Nef. Proteomic analysis in HEK293T cells indicated that 93 proteins were upregulated and 232 were downregulated in their ubiquitination status by Nef. Computational analysis classified these proteins based on molecular function, biological process, subcellular localization, and biological pathway. Of those proteins, we found a majority of molecular functions to be involved in binding and catalytic activity. With respect to biological processes, a significant portion of the proteins identified were related to cellular and metabolic processes. Subcellular localization analysis showed the bulk of proteins to be localized to the cytosol and cytosolic compartments, which is consistent with the known function and location of Nef during HIV-1 infection. As for biological pathways, the wide range of affected proteins was denoted by the multiple modes to fulfill function, as distinguished from a strictly singular means, which was not detected. Among these ubiquitinated proteins, six were found to directly interact with Nef, wherein two were upregulated and four downregulated. We also identified 14 proteins involved in protein stability through directly participating in the Ubiquitin Proteasome System (UPS)-mediated proteasomal degradation pathway. Of those proteins, we found six upregulated and eight downregulated. Taken together, these analyses indicate that HIV-1 Nef is integral to regulating the stability of various cellular proteins *via* modulating ubiquitination. The molecular mechanisms directing Nef-triggered regulation of cellular protein ubiquitination are currently under investigation.

## 1 Introduction

It is reported that HIV-1 Nef alone is capable of inducing adult acquired immune disorder syndrome (AIDS)-like diseases in CD4C/HIV(nef) transgenic mice (Hanna et al., 1998; Rahim et al., 2009). Long-term survivors with nonprogressive HIV-1 infection can carry crippled Nef (Deacon et al., 1995; Kirchhoff et al., 1995; Salvi et al., 1998), and destruction of the *nef* gene in SIV can preclude simian AIDS in macaque monkeys (Kestler et al., 1991). These findings indicate that Nef could be essential for efficient replication *in vivo* and induction of AIDS in both HIV-1- and SIV-infected adult hosts (Kestler et al., 1991; Deacon et al., 1995; Kirchhoff et al., 1995; Salvi et al., 1998). However, since Nef is generally known to be dispensable for *in vitro* replication of both HIV-1 and most SIVs (Gibbs et al., 1994; Park and Sodroski, 1995; Stoddart et al., 2003; Rainho et al., 2015), the cellular and molecular effects of Nef expression on HIV-1/SIV replication, especially in CD4+ T cells, have been essentially immeasurable. Thus, it has been challenging to determine the role of Nef in virus-associated pathogenicity.

The function of HIV-1 Nef is multifarious, as the protein plays a critical role in: T cell activation for virus replication (Sleckman et al., 1992; Salghetti et al., 1995; Brown et al., 1999; Kim et al., 1999; Schibeci et al., 2000; Wang et al., 2000); membrane trafficking of molecules, such as CD4 (Guy et al., 1987; Garcia and Miller, 1991; Anderson et al., 1993; Mariani and Skowronski, 1993; Rhee and Marsh, 1994), major histocompatibility complex class (MHC I) (Schwartz et al., 1996; Le Gall et al., 1997), and others (Aiken et al., 1994; Schwartz et al., 1996) to facilitate HIV-1 infectivity and immune escape; stability of viral and cellular proteins *via* the ubiquitin proteasome system (Sugiyama et al., 2011; Kmiec et al., 2018; Pyeon et al., 2019; Ali et al., 2020; Qiu et al., 2020; Zhang et al., 2020); chemotaxis (Swingler et al., 1999; Choe et al., 2002; Park and He, 2009; Stolp et al., 2009; Stolp et al., 2012; Park and He, 2013; Rossi et al., 2016; Lamas-Murua et al., 2018); and intercellular communications through exosomes (Lenassi et al., 2010; Narayanan et al., 2013; McNamara et al., 2018; Mukhamedova et al., 2019; Chen et al., 2020; Dubrovsky et al., 2020) and conduits/filopodia (Xu et al., 2009; Nobile et al., 2010; Tan et al., 2013; Park et al., 2014). Molecular processes, such as signaling cascades, gene expression, etc., leading to these biological changes by HIV-1 Nef have been comprehensively investigated, as reviewed (Renkema and Saksela, 2000; Abraham and Fackler, 2012; Felli et al., 2017; Heusinger and Kirchhoff, 2017; Buffalo et al., 2019; Staudt et al., 2020).

In addition to the above-noted Nef functions, several recent reports suggest that Nef plays an integral role in the determination of HIV-1-mediated pathogenicity by modulating stability of viral and cellular proteins essential for viral replication. Substitution of all the lysine residues in Nef with arginine abolished Nef ubiquitination and subsequently CD4 downregulation (Jin et al., 2008), indicating that Nef ubiquitination is important for modulating the surface expression of CD4. Degradation of Nef is mediated by interaction with c-Cbl, a host ubiquitin (Ub) E3 ligase (Zhang et al., 2020), while our previous reports show that UBE3A (E6-AP), another host Ub E3 ligase, determines stability of Nef (Pyeon et al., 2019). Nef can also promote proteasomal degradation of host cellular proteins, such as p53 tumor suppressor using UBE3A (Ali et al., 2020) and CXCR4 by recruiting the HECT domain E3 ligases, AIP4 or NEDD (Chandrasekaran et al., 2014). Moreover, Nef regulates the degradation of a key HIV-1 viral transcription activator protein, Tat. It is reported that Nef spurs decay of Tat for HIV-1 replication *via* the ubiquitin proteasome system (UPS) (Sugiyama et al., 2011), indicating that Nef could function as a global regulator for viral gene expression by modulating the intracellular level of Tat. Taken together, these reports indicate that Nef plays a crucial role in coordinating the stability of viral and cellular proteins *via* the UPS in infected cells, which is essential for HIV-1 replication followed by HIV-1 pathogenesis.

We have demonstrated that HIV-1 Nef binds to ubiquitin protein ligase E3A (UBE3A) by the yeast two-hybrid system using Jurkat cDNA library followed by immunoprecipitation/Western blot and that UBE3A degraded not only Nef but also HIV-1 structural proteins, Gag, thus significantly inhibiting HIV-1 replication only in the presence of Nef in HIV-1 susceptible Jurkat T cells (Pyeon et al., 2019). We further found that Nef inhibited the level of ubiquitination of cellular proteins and that mutations at specific motifs in Nef significantly reduced the Nef-mediated inhibitory effects on ubiquitination of cellular proteins (Pyeon et al., 2019). While it is evident that Nef regulates cellular protein ubiquitination, these investigations did not reveal the specific cellular proteins ubiquitinated by Nef. Therefore, we executed proteomic analysis to identify differentially ubiquitinated cellular proteins in the presence of Nef, which brings insights into the pathogenic role of Nef in HIV-1 infected cells.

## 2 Materials and methods

### 2.1 Cells, antibodies and plasmids

Human Embryonic Kidney (HEK) 293T cells were cultured in Dulbecco modified Eagle medium (DMEM) complemented with 10% fetal bovine serum (FBS) growth supplement and 1% penicillin/streptomycin in a humidified 37° C/5% CO_2_ incubator. HIV-1 Nef-expressing plasmid (pHN) tagged with a Myc.His epitope was generated by cloning the nef coding region of HXBc2 strain HIV-1 into the EcoRI and BamHI sites of pCDNA3.1(−)-Myc.His (pC3) (Agilent, Santa Clara, CA, USA). The native pC3 plasmid was used as a backbone control such that the total amount of the plasmid was the same for as pHN transfections. HA epitope-tagged ubiquitin-expressing plasmid (pUb-HA) was obtained through Addgene (Cambridge, MA, USA). Anti-Myc (9E10) and anti−HA (F-7) antibodies were purchased from Santa Cruz (Santa Cruz, CA, USA). Manufacturers and catalogue numbers of other antibodies that we employed for these experiments are; SMAD3 (ABclonal, A11388), E6-BP (Santa Cruz, sc-293069), PAXBP1 (Bethyl Laboratory, A303-166A), KEAP (Invitrogen, 1F10B6), ATIC (Invitrogen, PA5-827411), and β-Actin (Sigma-Aldrich, A5441).

### 2.2 Immunoprecipitation (IP) and western blot (WB) analysis

HEK 293T cells were transfected with the indicated plasmids through the calcium phosphate method. Forty-eight hours post-transfection, the cells were washed in ice-cold PBS twice prior to suspension in lysis buffer (50 mM Tris-HCl pH 7.4, 300 mM NaCl, 1% NP-40, 50 mM NaF, 1 mM Na_3_VO_4_, 1 mM PMSF and 1× protease inhibitor cocktail (Calbiochem, La Jolla, CA, USA)). Following 20 minutes of incubation on ice and 20 minutes of centrifugation at 20,000× g at 4°C, the cell lysate supernatant was collected and used for WB analysis as previously described (Park et al., 2013). IP was performed using anti−HA antibody to precipitate ubiquitinated proteins in the supernatants, and the precipitated proteins were separated on sodium dodecyl sulfate–polyacrylamide gel electrophoresis (SDS-PAGE) by running approximately 1 cm. The proteins on SDS-PAGE were then stained with Coomassie Blue, and the proteins were extracted for proteomic analysis. For the WB analysis, the horseradish peroxidase (HRP)-conjugated secondary antibodies were purchased from Thermo-Fisher and enhanced chemiluminescence (ECL) from Bio-Rad, as described [57]. The IP and/or WB analyses in the figures are representative of multiple independent experiments.

### 2.3 Proteomic analysis

Following SDS-PAGE, the ubiquitinated proteins were extracted and analyzed by the combined high-affinity enrichment of ubiquitinated peptides and high-resolution liquid chromatography tandem mass spectrometry (LC-MS/MS) (10.1016/j.jprot.2021.104261; 10.3389/fpls.2018.01064). The fold-changes in ubiquitination of the differentially ubiquitinated cellular proteins were calculated using ratios comparing ubiquitination in the presence and absence of Nef. Cellular proteins with a fold-change in ubiquitination greater than two were considered significantly differentially ubiquitinated and included in subsequent analyses. Analysis and classification of these cellular proteins by function, biological process, cellular component, pathway, and protein class were achieved using the Protein ANalysis THrough Evolutionary Relationships (PANTHER) Classification system (16.0) (http://www.pantherdb.org) (Mi et al., 2021). The STRING Database (STRING-DB) was used to conduct protein-protein interaction network and functional enrichment analysis of the identified cellular proteins (https://string-db.org/) (Szklarczyk et al., 2019). The magnitudes of identified enrichment effects were represented by strength scores, log10(observed number of proteins in the given network with a particular characteristic/expected number of proteins in a random network of the same size with the given characteristic). Significance of enrichment was assessed using false discovery rate p-values, which were corrected for multiple testing within each characteristic category using the Benjamini-Hochberg procedure. Additional protein interaction data was collected using the National Institutes of Health (NIH) GENE database (https://www.ncbi.nlm.nih.gov/gene) (Brown et al., 2015) and the WikiPathways database (https://www.wikipathways.org/), which was specifically used to collect data on protein interactions with the ubiquitin-proteasome (UPS)-mediated proteasomal degradation pathway (Slenter et al., 2018). Tableau Public (version 2020.3) (http://www.tableau.com) was used to create visualizations presented in the figures.

## 3 Results

### 3.1 Nef reduces overall ubiquitination of cellular proteins

**Figure 1:**
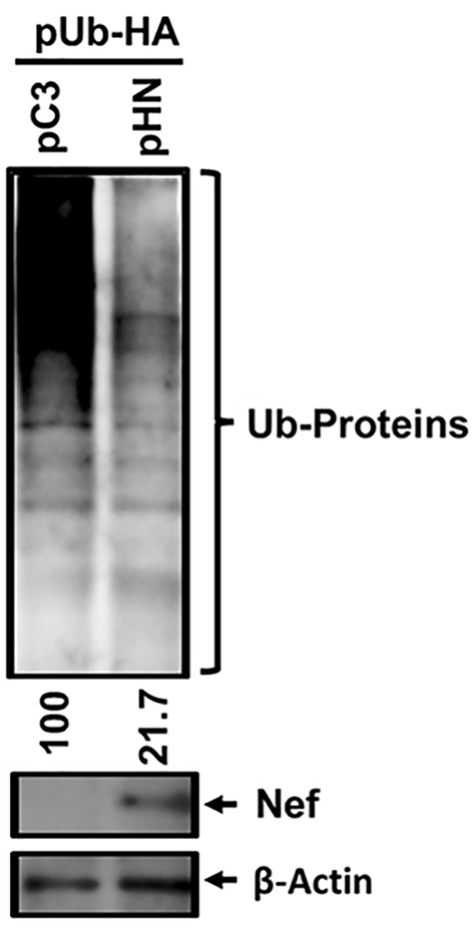
Effect of HIV-1 Nef on overall ubiquitination of cellular proteins. The indicated plasmids, pUb-HA and pHN-Myc.His (pHN) or pCDNA3.1(−)-Myc.His (pC3), were transfected into 293T cells, and 3 days post-transfection, WB analysis was executed with the cell lysates generated from the transfected cells to detect the indicated proteins. The relative intensity of the ubiquitinated cellular proteins (Ub-Proteins) in the presence (47.6) compared with the absence of Nef (100) was determined based on normalization of β-Actin, using Bio-Rad image analysis tool.

To investigate the effect of HIV-1 Nef on the ubiquitination status of cellular proteins in host cells, we co-transfected pUb-HA and the pHN-Myc.His plasmids into HEK 293T cells. An isotype plasmid lacking Nef (pCDNA3.1(−)-Myc.His), was also included as a backbone control instead of pHN-Myc.His. The subsequent WB analysis of cell lysates using anti-HA antibodies to detect ubiquitinated cellular proteins showed that the relative intensity of ubiquitinated cellular protein bands was lower in cells expressing HIV-1 Nef compared with the backbone control. The relative band intensities were 21.7 to 100, respectively, when normalized to β-Actin as a protein loaded control (**Figure 1**). Further, deletion of the di-arginine (Di-R) motif of Nef expunged Nef-mediated inhibition of ubiquitination of cellular proteins (Pyeon et al., 2019). These data indicated that overall HIV-1 Nef inhibited the ubiquitination of cellular proteins in 293T cells.

### 3.2 The presence of Nef results in upregulation of ubiquitination in 93 cellular proteins and downregulation in 232

**Figure 2:**
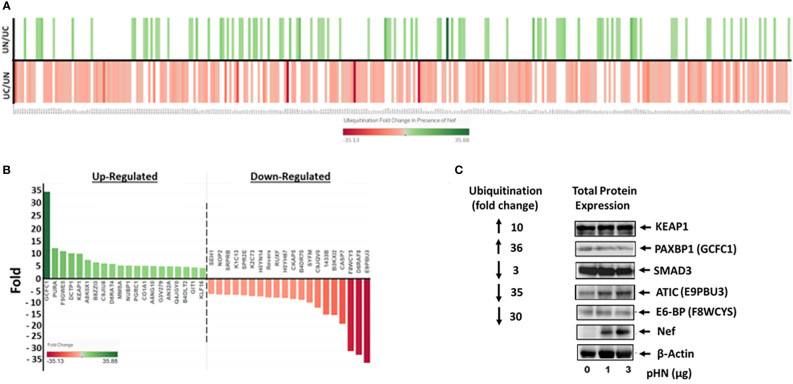
HIV-1 Nef-mediated differential ubiquitination of individual cellular proteins. **(A)** Heatmap showing Nef-mediated ubiquitination fold change of cellular proteins. The identified 325 cellular proteins that are differentially ubiquitinated in the presence of Nef with statistical significance are labeled along the X-axis, while increases and decreases of ubiquitination of cellular proteins in the presence (UN) and absence (UC) of Nef, respectively, are depicted on the Y-axis; that is, increases in the presence of Nef (UN/UC) are shown by a green line, whereas decreases in ubiquitination in the presence of Nef are indicated by a red line in the UC/UN. Color intensities of each line correspond to fold changes in ubiquitination of the indicated cellular proteins. **(B)** Top 20 positive and negative ubiquitination fold changes of cellular proteins in the presence of Nef. **(C)** Validation of Nef-mediated differential ubiquitination of cellular proteins. The level of expression of the indicated proteins in the presence of Nef was nominal, while that of ubiquitination of the proteins was profoundly increased (KEAP1 and PAXBP1) or decreased (SMAD3, ATIC, and E3-BP), indicating that changes in ubiquitination of these proteins were not due to the expression of each protein. Parentheses represent aliases of the indicated genes in part label **(B)**.

Following WB analysis, proteomic analysis identified differentially ubiquitinated cellular proteins in the presence of Nef. The data showed statistical significance in 325 cellular proteins that differentially ubiquitinated in the presence of Nef. Among them, ubiquitination of 93 cellular proteins was significantly upregulated, while that of 232 proteins was down-regulated, as shown in **Figure 2A**. Fold changes in ubiquitination of these proteins ranged from a 35.88-fold increase to a 35.13-fold decrease. The 20 cellular proteins with the greatest fold increases and 20 proteins with the greatest fold decreases in ubiquitination are highlighted in **Figure 2B**.

To validate these ubiquitination results, several proteins involved in crucial cellular processes were analyzed by western blot to compare Nef induced changes in total protein expression to their relative changes in ubiquitination. HEK293T cells were transfected with three different dosages (0 µg, 1 µg, and 3 µg) of pHN, the HIV-1 Nef expression plasmid (**Figure 2C**). We first evaluated KEAP1 (the E3 ligase adaptor Kelch-like ECH-associated protein 1), critical in the maintenance of redox, metabolic and protein homeostasis, and the regulation of inflammation (Cuadrado et al., 2019; Dayalan Naidu and Dinkova-Kostova, 2020). Expression of KEAP1 was basically unchanged by Nef, while ubiquitination of KEAP1 was increased 10-fold in the presence of Nef (**Figures 2B, C**). The protein expression of PAXBP1, a transcription regulator (Zhang et al., 2000), was reduced marginally as Nef expression increased. Yet, HIV-1 Nef-associated ubiquitination of PAXBP1 increased 36-fold (**Figures 2B, C**). Expression of SMAD3, which is involved in TGF-β cytokine signal transduction and regulation of cell division, differentiation, and apoptosis (Massague, 1998) was unchanged by Nef expression, but ubiquitination of the protein decreased three-fold in the presence of Nef. The level of ATIC (5-Aminoimidazole-4-carboxamide ribonucleotide formyltransferase/IMP cyclohydrolase catalyzing enzyme in the *de novo* purine biosynthetic pathway (Sugita et al., 1997)), which is known to facilitate cell growth and migration by upregulating Myc expression in lung adenocarcinoma (Niu et al., 2022), was slightly increased, as Nef expression was increased (**Figure 2C**). In contrast, ubiquitination of the protein was dramatically reduced by 35-fold in the presence of Nef (**Figure 2B**). Lastly, E6-BP, a calcium-binding protein relevant to the carcinogenic potential of HPV E6 (Chen et al., 1995; Lichtig et al., 2006; Vazquez-Ortiz et al., 2007), had marginally decreased in expression as Nef expression increased, yet ubiquitination in the presence of Nef decreased 30-fold. Together, these representative proteins (**Figure 2C**) demonstrate that changes observed in ubiquitination levels were indeed associated with differential ubiquitination of individual proteins, rather than Nef induced changes in their overall expression levels. Further, these profound fold changes in ubiquitination of numerous cellular proteins with diverse cellular/molecular functions shown below suggest that Nef plays a pivotal role in the regulation of ubiquitination of cellular proteins and thereby cellular protein stability and decay through the UPS in HIV-1-infected cells to establish productive infection.

### 3.3 Several differentially ubiquitinated cellular proteins in the presence of Nef interact directly with Nef and the UPS

**Figure 3:**
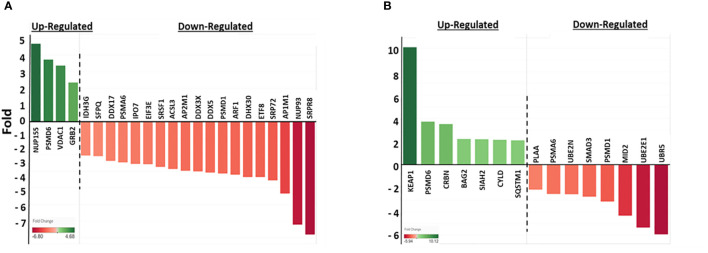
Nef-mediated changes in ubiquitination of cellular proteins that directly interact with Nef or the UPS. **(A)** Nef-mediated ubiquitination fold change in proteins that directly interact with Nef. Twenty-three of the 325 cellular proteins identified in **Figure 2A** directly interact with Nef, according to the data collected from the NIH GENE database (https://www.ncbi.nim.nih.gov/gene). The fold changes in ubiquitination in the presence of Nef range from 6.8-fold decrease to a 4.68-fold increase, with greater color intensity corresponding to larger fold changes. The fold changes associated with cellular proteins for which Nef up-regulated ubiquitination are depicted in green, while those with down-regulated ubiquitination are in red. **(B)** Nef-mediated ubiquitination fold changes in proteins that directly interact with the UPS. Fifteen of the 325 cellular proteins identified in **Figure 2A** directly interact with UPS, according to the data collected from the WikiPathways database (https://www.wikipathways.org/). The fold changes in ubiquitination range from a 5.94-fold decrease to a 10.12-fold increase. The same color coding was used in both panels **(A, B)**.

To establish how Nef regulates ubiquitination, we searched the NIH GENE database to determine if Nef is known to physically interact with any of the 325 differentially ubiquitinated cellular proteins. Our analysis indicated that Nef directly forms complexes with 23 cellular proteins with ubiquitination fold changes ranging from a 4.68-fold increase to a 6.8-fold decrease (**Figure 3A**), demonstrating that Nef may play an important role in modulating stability of cellular proteins by direct interactions with the indicated cellular proteins within HIV-1-infected host cells. Understanding the implications of direct and indirect relationships between Nef and host proteins may provide further insight into the roles of Nef in HIV-1-host interactions. Since we hypothesize that Nef regulates ubiquitination and cellular protein stability through the UPS, the WikiPathways database was used to determine whether any of the 325 cellular proteins were functionally associated with the UPS. We found that 15 of the proteins directly interact with the UPS, with ubiquitination fold changes ranging from a 10.12-fold increase to a 5.94-fold decrease (**Figure 3B**). These findings further support our conclusion that Nef is a critical regulator of cellular protein stability by modulating the level of ubiquitination of the UPS degradation pathway *via* direct and indirect associations with the cellular proteins.

### 3.4 Differentially ubiquitinated cellular proteins significantly localize to cellular compartment and tissues where Nef expression has been observed and have key cellular functions, including regulation of gene expression, the cell cycle, and cell migration

**Figure 4:**
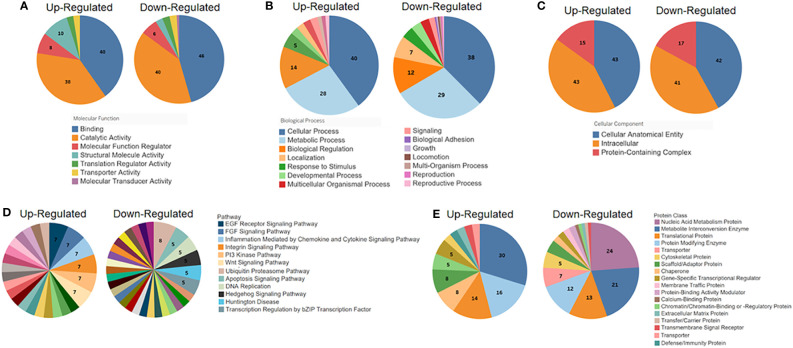
PANTHER classification (http://pantherdb.org) analysis of the significantly differentially ubiquitinated proteins by **(A)** molecular function, **(B)** biological process, **(C)** cellular localization, **(D)** pathway, and **(E)** protein class. For each set of classifications, proteins with up-regulated ubiquitination in the presence of Nef (left graph in each set) were analyzed and presented separately from those with down-regulated ubiquitination (right graph in each set).

To characterize the 325 cellular proteins differentially ubiquitinated by Nef, the PANTHER classification system was employed to categorize the proteins by molecular function, biological process, cellular localization, pathway, and protein class. The classifications of cellular proteins were highly similar regardless of whether ubiquitination was up- or down-regulated by Nef (**Figures 4A–C**). In terms of molecular functions, the proteins primarily played binding or catalytic roles (**Figure 4A**). More specifically, most of the identified binding proteins play roles in protein, heterocyclic compound, and organic cyclic compound binding, while the catalytic proteins act on other proteins or function as hydrolases or transferases. A majority of the ubiquitinated proteins also function in cellular and metabolic processes (**Figure 4B**), especially in cellular component organization, biogenesis, and metabolism of macromolecules, aromatic compounds, or nitrogen compounds. Classification of the cellular localizations of the ubiquitinated proteins indicated that over 80% localize to cellular anatomical entities, particularly the cytoplasm, cellular membrane, and organelles, or to intracellular spaces, as well as particularly catalytic or ribonucleoprotein complexes (**Figure 4C**). These localization patterns are consistent with the previously established localization patterns of Nef during HIV-1 infection (Trible et al., 2006; Atkins et al., 2008).

Pathway and protein class classifications exhibited more differences between proteins with upregulated and downregulated ubiquitination. The proteins, whether up- or down-regulated in ubiquitination, represent involvement in a wide variety of pathways. The most common pathways of proteins with upregulated ubiquitination were chemokine and cytokine signaling pathways (7%), which mediate inflammation, and the epidermal growth factor (EGF) receptor pathway (7%) and fibroblast growth factor (FGF) signaling pathway (7%), which both regulate cell division, differentiation, proliferation, and death (Oda et al., 2005; Teven et al., 2014) (**Figure 4D**). For proteins downregulated in ubiquitination, most were UPS pathway proteins (8%), consistent with earlier conclusions that Nef may regulate protein stability through mechanisms involving this pathway. The apoptosis signaling pathway and DNA replication pathways were among the subsequent most common pathways observed (5% each, respectively). Lastly, over half of proteins with upregulated ubiquitination were classified as metabolite interconversion enzymes (30%), protein modifying enzymes (16%), or translational proteins (14%) (**Figure 4E**). Sizable proportions of proteins with downregulated ubiquitination were categorized as nucleic acid metabolism proteins (24%), metabolite interconversion enzymes (21%), translational proteins (13%), or protein modifying enzymes (12%), showing some overlapping patterns with those proteins which were upregulated in their ubiquitination status.

Further details regarding classification and associated statistical significances of the differentially ubiquitinated proteins were elucidated through functional enrichment analysis using STRING-DB. Of the 93 proteins with significantly increased ubiquitination in the presence of Nef, 53 could be located within the database. Analysis of proteins with upregulated ubiquitination in the presence of Nef showed significant functional enrichment in 13 cellular components and 16 tissues. No significant pathway enrichments were observed in the biological process, molecular function, Reactome pathway, or disease-gene association (**Table 1**). Cellular component classification confirmed the patterns identified through PANTHER analysis, with significant enrichment of cytoplasm (strength=0.17, p=0.0042), intracellular organelle lumen (strength=0.28, p=0.0150, intracellular membrane-bounded organelle (strength=0.16, p=0.0256), intracellular organelle (strength=0.14, p=0.0150), membrane-bounded organelle (strength=0.14, p=0.0200), organelle (strength=0.14, p=0.0042), intracellular localization (strength=0.13, p=0.0020), and protein-containing complexes (strength=0.34, p=0.0020) (**Table 1**). However, the analysis revealed that the strongest significant enrichment effects were associated with protein localization to actin caps (strength=2.27, p=0.0181), lamellipodia (strength=0.96, p=0.0268) (**Table 1**), and focal adhesions (strength= 0.80, p=0.0181), suggesting that differential ubiquitination of the proteins in the presence of Nef may indirectly impact cell migration, which is a process that these three cellular components play key roles in (Ridley et al., 2003; Kim et al., 2014; Innocenti, 2018). Regarding tissue expression, the most significant enrichment was identified in lung (strength= 0.65, p=0.0018), respiratory system (strength=0.59, p=0.0020), skin (strength=0.58, p=0.0177), and alimentary canal (strength=0.47, p=0.0176) tissues (**Table 1**), all of which are tissues in which Nef has been detected in subjects with HIV-1 (Marecki et al., 2006; Quaranta et al., 2011).

**Table 1:** String-DB (https://string-db.org/) functional enrichment analysis of cellular proteins with increased ubiquitination in the presence of Nef identified significant enrichment in 13 cellular components and 16 tissues, based on the 53 out of 93 proteins identified in the database.

Cellular Component (Gene Ontology)			
description	count in network	strength	false discovery rate
Actin cap	2 of 4	2.27	0.0181
Lamellipodium	5 of 202	0.96	0.0268
Focal adhesion	7 of 405	0.8	0.0181
Protein-containing complex	30 of 5073	0.34	0.0020
Cytosol	28 of 5193	0.3	0.0150
Intracellular organelle lumen	30 of 5857	0.28	0.0150
Nucleus	33 of 7390	0.22	0.0281
Cytoplasm	46 of 11428	0.17	0.0042
Intracellular membrane-bounded organelle	42 of 10761	0.16	0.0256
Organelle	50 of 13515	0.14	0.0042
Intracellular organelle	47 of 12528	0.14	0.0150
Membrane-bounded organelle	46 of 12427	0.14	0.0200
Intracellular	52 of 14276	0.13	0.0020
Tissue expression (TISSUES)			
description	count in network	strength	false discovery rate
Lung	14 of 1162	0.65	0.0018
Respiratory system	15 of 1436	0.59	0.0020
Skin	11 of 1069	0.58	0.0177
Alimentary canal	15 of 1865	0.47	0.0176
Internal female genital organ	20 of 2593	0.45	0.0027
Integument	15 of 1970	0.45	0.0232
Digestive gland	19 of 2645	0.42	0.0100
Organism form	16 of 2239	0.42	0.0257
Embryonic structure	15 of 2132	0.41	0.0427
Viscus	32 of 5020	0.37	0.00020
Endocrine gland	33 of 6036	0.3	0.0018
Reproductive system	31 of 6107	0.27	0.0100
Nervous system	28 of 5707	0.26	0.0314
Urogenital system	32 of 6716	0.25	0.0176
Female reproductive system	28 of 5799	0.25	0.0395
Animal	51 of 14895	0.1	0.0173

The “count in network” column shows how many of the proteins in the 53-protein network are associated with a given term, out of the total expected proteins across the entire genome associated with the term. Strength quantifies the enrichment effect size and is calculated using the formula: log10(observed/expected), where “observed” is the number of proteins in the network associated with a given term and “expected” is the number of proteins expected to be associated with a given term in a random network of the same size. The false discovery rate is the p-value representing the enrichment’s significance and is corrected for multiple testing through the Benjamini-Hochberg procedure.

Of the 232 proteins with significantly decreased ubiquitination in the presence of Nef, 140 were located within STRING-DB. Functional enrichment analysis of these proteins identified significant functional enrichment in 35 cellular components, 48 tissues, 97 biological processes, 26 molecular functions, and 24 Reactome pathways. No significant enrichment was observed in disease-gene associations (**Table 2**). Among the cellular component localizations with the strongest enrichment were cargo-selective retromers (strength=1.84, p=0.0339) and tubular endosomes (strength=1.75, p=0.0437), which play key roles in intracellular trafficking (Harbour et al., 2010), a cellular process that Nef is known to interfere with (Pereira and daSilva, 2016) (**Table 2**). For tissue expression, significant enrichment was also observed in B-cell lymphoma cells (strength=0.84, p=0.0035), lymphoblasts (strength=0.82, p=8.21e-05), lymphoma cells (strength=0.74, p=0.0052), colonic cancer cells (0.00079), and brain cells (strength=0.76, p=0.0266). These enrichments also align with prior studies demonstrating that Nef plays roles in lymphoma and colon cancer development (Bumpers et al., 2005) and is highly expressed in astrocyte brain cells (Sami Saribas et al., 2017) (**Table 2**). Regarding Reactome pathways, significant enrichment of proteins involved in DNA synthesis and repair, specifically lagging strand synthesis (strength=1.32, p=0.0451) and PCNA-dependent long patch base excision repair (strength=1.3, p=0.0494), was identified (**Table 2**).

**Table 2:** String-DB (https://string-db.org/) functional enrichment analysis of cellular proteins with decreased ubiquitination in the presence of Nef identified significant enrichment in 35 cellular components, 48 tissues, 97 biological processes, 26 molecular functions, and 24 Reactome pathways, based on the 140 out of 232 proteins identified in the database.

Cellular Component (Gene Ontology)			
description	count in network	strength	false discovery rate
Retromer, cargo-selective complex	2 of 4	1.84	0.0339
Tubular endosome	2 of 5	1.75	0.0437
DNA replication factor C complex	2 of 5	1.75	0.0437
Eukaryotic 48s preinitiation complex	3 of 15	1.45	0.0165
Eukaryotic translation initiation factor 3 complex	3 of 15	1.45	0.0165
Eukaryotic 43s preinitiation complex	3 of 17	1.39	0.0194
Vesicle coat	5 of 53	1.12	0.0040
Catalytic step 2 spliceosome	7 of 87	1.05	0.00041
Spliceosomal complex	10 of 192	0.86	0.00018
Condensed chromosome kinetochore	5 of 106	0.82	0.0467
Kinetochore	6 of 134	0.8	0.0248
Condensed chromosome	7 of 216	0.66	0.0441
Ribonucleoprotein complex	21 of 677	0.64	2.59e-06
Spindle	10 of 353	0.6	0.0165
Nuclear speck	11 of 399	0.59	0.0116
Tissue expression (TISSUES)			
description	count in network	strength	false discovery rate
B-cell lymphoma cell	7 of 140	0.84	0.0035
Lymphoblast	11 of 235	0.82	8.21e-05
Brain cell line	6 of 145	0.76	0.0266
Colonic cancer cell	10 of 249	0.75	0.00079
Lymphoma cell	8 of 203	0.74	0.0052
Fibroblast	7 of 213	0.66	0.0355
Liver	60 of 1882	0.65	5.65e-22
Digestive gland	78 of 2645	0.62	3.53e-28
Blood cancer cell	32 of 1098	0.61	1.17e-09
Lung	31 of 1162	0.57	2.14e-08
Leukemia cell	25 of 949	0.57	1.62e-06
Lymphocyte	16 of 648	0.54	0.00088
Skin	26 of 1069	0.53	3.73e-06
Embryo	17 of 708	0.53	0.00077
Respiratory system	34 of 1436	0.52	4.94e-08

The strongest 15 enrichments for each category are displayed in the table. The “count in network” column shows how many of the proteins in the 53-protein network are associated with a given term, out of the total expected proteins across the entire genome associated with the term. Strength quantifies the enrichment effect size and is calculated using the formula: log10(observed/expected), where “observed” is the number of proteins in the network associated with a given term and “expected” is the number of proteins expected to be associated with a given term in a random network of the same size. The false discovery rate is the p-value representing the enrichment’s significance and is corrected for multiple testing through the Benjamini-Hochberg procedure.

Finally, analysis of the proteins’ biological processes and functions corroborated and expanded upon patterns observed in the PANTHER analysis, with binding, metabolic, and translation-related activities being highly enriched. Specifically, the analysis detected strong enrichment effects for molecular functions including RNA helicase activity (strength=1.05, p=0.0038), ribonucleoprotein complex binding (strength=0.98, p=0.00032), RNA-binding translation factor activity (strength=0.93, p=0.0451), nucleic acid-binding translation regulator activity (strength=0.92, p=0.0179), and RNA binding (strength=0.65, p=5.46e-18). The strongest enrichment effects for biological processes were observed in tRNA N2-guanine methylation (strength=2.15, p=0.342), hexitol metabolism (strength=1.97, p=0.0496), mature ribosome assembly (strength=1.84, p=0.0053), and regulation of mRNA binding (strength=1.75, p=0.00069) (**Table 2**).

**Figure 5:**
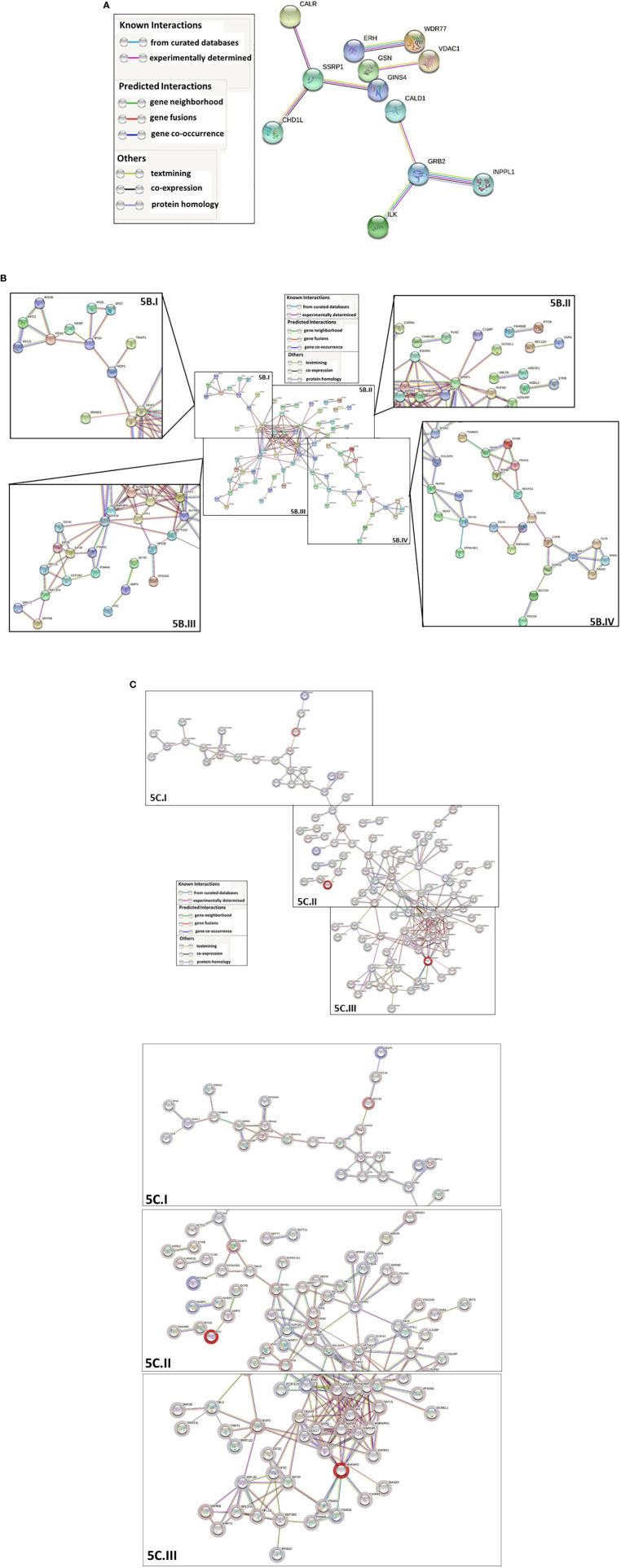
STRING-DB (https://string-db.org/) protein-protein interaction network analysis of the proteins that are differentially ubiquitinated in the presence of Nef. For all three analyses, a high confidence minimum required interaction score (0.7) was specified, only query protein nodes were displayed, and disconnected nodes were hidden. Network edges represent predicted protein-protein functional associations and are color-coded by evidence type, as outlined in the associated legends. The whole genome was used as the background for the analysis. For parts **(B, C)**, networks were shown in their entirety, then segmented into the components labeled with Roman numerals to increase legibility of individual protein names. **(A)** Network analysis of proteins with significantly upregulated ubiquitination. 53 of the 93 proteins with significantly upregulated ubiquitination were identified in the database. The final network contained 53 nodes and 8 edges, with 8 expected edges and a nonsignificant PPI enrichment p-value of 0.516. **(B)** Network analysis of proteins with significantly downregulated ubiquitination. 140 of the 232 proteins with significantly downregulated ubiquitination were identified in the database. The final network contained 140 nodes and 166 edges, with 74 expected edges and a significant PPI enrichment p-value < 1.0e-16. The network was segmented into four zoomed-in components labeled with Roman numerals (I-IV) so that individual protein names could be legible. **(C)** Network analysis of all proteins with differentially ubiquitination in the presence of Nef. The final overall network contained 193 nodes and 247 edges, with 131 expected edges and a significant PPI enrichment p value < 1.0e-16. Node halos were colored along a gradient based on relative fold change in ubiquitination. Dark red represents the greatest fold decrease in ubiquitination and dark blue the greatest fold increase. The network was segmented into three zoomed-in components, labeled (I-III) so that individual protein names could be legible.

Protein-protein interaction network analysis using STRING-DB was conducted to further analyze the differentially ubiquitinated proteins. Analysis of proteins with upregulated ubiquitination showed 8 significant predicted functional associations within the set of 53 proteins present in the database; however, those interactions were not statistically significantly greater than would be expected in a random set of proteins with the same size and degree distribution (protein-protein interaction (PPI) enrichment value=0.516; high confidence (0.7) minimum required interaction score) (**Figure 5A**). This finding is indicative of little to no significant biological relationship between the 53 proteins, but it is important to note that availability of data on several of the proteins’ interactions within the database is a limitation that may at least partially explain this outcome. Network analysis of the proteins with downregulated ubiquitination identified 166 statistically significant predicted functional associations between proteins within the 140-protein network (PPI enrichment value < 1.0e-16; 0.7 minimum required interaction score) (**Figure 5B**), suggesting a biological connection between the proteins. The average node degree was 2.37, meaning that each of the proteins has an average of 2.37 interactions within the network (0.7 minimum required interaction score). Finally, when all 193 differentially ubiquitinated proteins found in the database were analyzed together, 247 predicted functional associations were found, with an average node degree of 2.56 and a significant PPI enrichment p-value < 1.0e-16 (0.7 minimum required interaction score) (**Figure 5C**). This demonstrates that proteins that are up- and down-regulated in ubiquitination interact and that the proteins that are differentially ubiquitinated in the presence of Nef share biological characteristics and connections, rather than being a random grouping of proteins.

Overall, our PANTHER classification analysis showed that most of the differentially ubiquitinated cellular proteins are involved in binding and catalytic activities, participate in metabolic processes, and localize to cellular regions where Nef is usually observed. Additionally, several fall into protein classes or are involved in pathways associated with the UPS, gene regulation, cellular division, proliferation, and death. The STRING-DB enrichment analyses expanded on these results, demonstrating that cellular proteins with up-regulated ubiquitination in the presence of Nef significantly localize to the cytoplasm, organelle, and cell migration-associated structures within cells, with especially enriched presence in respiratory and epithelial cells. Cellular proteins displaying down-regulated ubiquitination in the presence of Nef displayed strong localization to intracellular trafficking-related cellular structures and lymphoma tissues, with highly enriched activity in transcription regulation, metabolism, and RNA, nucleic acid, and ribonucleoprotein binding. Lastly, network analysis indicated that, in addition to sharing similar characteristics such as localization and function, many cellular proteins that are differentially ubiquitinated in the presence of Nef significantly interact with one another and are at least partially biologically connected. Taken together, these findings suggest that Nef-mediated differential ubiquitination of cellular proteins may have significant implications for the regulation of cellular protein stability, thereby indirectly influencing key cellular processes, such as metabolism and gene and cell cycle regulation.

## 4 Discussion

Consistent with our previous data [28], HIV-1 Nef clearly decreases the overall ubiquitination level of cellular proteins in Nef-expressing cells. In fact, Nef directly associates with numerous cellular proteins whose ubiquitination status was modulated by Nef expression (**Figure 3A**), and many of these proteins are involved in proteasomal degradation processes (**Figure 3B**). Further, differentially ubiquitinated cellular proteins regulated by Nef were involved in metabolic processes and in protein binding with catalytic activities. The protein localization was to specific subcellular compartments, such as cytoplasm, cell membrane, and so forth, where Nef is usually detected. These associations help to highlight the fidelity and significance of our proteomic ubiquitination analysis. Prior study has shown that, in the presence of the proteasome inhibitor MG132, Nef-mediated effects on proteasomal degradation activity were negated, suggesting that Nef plays a crucial role in regulating cellular protein stability through this pathway (Pyeon et al., 2019). Taken together, our extensive analyses indicate that HIV-1 Nef is vital to the regulation of cellular protein stability *via* differential ubiquitination of proteins, and is thus at the foundation of Nef-associated pathogenicity in HIV-1-infected cells.

As discussed above, the functions of HIV-1 Nef are multifarious. Here, we highlight how Nef-associated changes in protein ubiquitination regulate many aspects of cellular and molecular function among many biological processes. Nef-mediated changes in the stability, availability and function of proteins can act in various cellular pathways affecting transcription, DNA replication, inflammation, cell division, differentiation, proliferation, and death. Although our findings contribute significantly to the understanding of Nef’s function, the intimate relevance of our *in vitro* Nef findings with respect to *in vivo* HIV-1 replication and its pathogenicity necessitates further study. Additionally, molecular mechanisms of Nef-triggered differential modulation of the ubiquitination status of these cellular proteins and the significance of regulating individual cellular proteins and pathways with respect to the reciprocal proliferation/restriction of HIV-1 infection will require further investigations to more fully understand the pathogenic role of Nef in HIV-1-infected cells.

## Data availability statement

The data supporting this study’s findings are available from the corresponding author upon reasonable request.

## Author contributions

Conceptualization and original draft preparation, I-WP; data acquisition, MG and I-WP; writing—review and editing, JP, MG, KB, and I-WP; supervision, KB and I-WP; funding acquisition, JP, MG, KB, and I-WP. All authors contributed to the article and approved the submitted version.
